# Testosterone Acts Within the Medial Amygdala of Rats to Reduce Innate Fear to Predator Odor Akin to the Effects of *Toxoplasma gondii* Infection

**DOI:** 10.3389/fpsyt.2020.00630

**Published:** 2020-07-02

**Authors:** Dhiraj Kumar Singh, Shantala Arundathi Hari Dass, Samira Abdulai-Saiku, Ajai Vyas

**Affiliations:** School of Biological Sciences, Nanyang Technological University, Singapore, Singapore

**Keywords:** androgen, behavioral manipulation, defensive behaviors, parasites, pheromones, semiochemicals

## Abstract

Rats infected with the protozoan *Toxoplasma gondii* exhibit a reduced aversion to cat odor. This behavioral change is thought to increase trophic transmission of the parasite. Infected male rats also show a greater testicular synthesis of testosterone and epigenetic change in arginine vasopressin within the medial amygdala. Here, we show that exogenous supply of testosterone within MeA of uninfected castrates recapitulates reduction in innate fear akin to behavioral change attributed to the parasite. We also show that castration post establishment of chronic infection precludes changes in fear and medial amygdala arginine vasopressin in the infected male rats. These observations support the role of gonadal hormones and pursuant neuroendocrine changes in mediating the loss of fear in the infected rats. This work also demonstrates that testosterone acting specifically within the medial amygdala sufficiently explains reduced defensive behaviors often observed during the appetitive component of reproductive behaviors.

## Introduction

Laboratory rats and mice infected with *Toxoplasma gondii* show reduced aversion to cat odors and an atypical attraction in a subset of animals (1, 2). This reduction in fear is assumed to increase predation by cats, who act as the definitive host for the parasite. Unequivocal evidence of greater predation for infected rats is still lacking (3). Nevertheless, loss of fear in this host-parasite relation presents a unique opportunity to study mechanisms of defensive behaviors. Several hypotheses have been advanced about proximate causation of reduced fear in this model (4–5). These possibilities can be grouped in two broad classes, including those that envision a central role of brain invasion by the parasite and those that argue that the brain invasion is merely incidental (4).

One among these hypotheses suggests that testicular invasion by *Toxoplasma gondii* initiates a neuroendocrine cascade (6). This invasion then leads to an increase in testosterone production and downstream epigenetic change in the medial amygdala of the brain, eventually leading to behavioral change due to the role of the medial amygdala in semiochemical processing (4, 7). Infection of rats with *Toxoplasma gondii* increases the amount of luteinizing hormone receptor and other rate-limiting enzymes involved in the synthesis of testosterone from its precursor in Leydig cells of the tests (6, 8). This causes an increase in circulating testosterone, which then crosses the blood-brain barrier and enhances transcription of arginine vasopressin (AVP) in the medial amygdala (7, 9). These neurons are part of the extra-hypothalamic vasopressin system and are important modulators of sociosexual behavior in male rodents (10–11). It is hypothesized that an increase in the tone of the medial amygdala vasopressin system reduces fear by increasing approach behavior (12), in line with the role of these neurons in facilitating reproductive behaviors which are often traded off with the defense. The important role of gonadal steroids is also supported by an increase in the production of major urinary proteins by male rats post-infection (13, 14), a phenotype that is testosterone-dependent (15). Moreover*, Toxoplasma gondii* infection increases behavioral impulsivity resembling effects produced by testosterone (16–17).

This leads to two unique predictions. Firstly, targeted testosterone supplementation within the medial amygdala should be sufficient to recapitulate behavioral change sans infection. Secondly, the removal of testes should lead to the rescue of host behavioral change. These predictions are in contrast to hypotheses built around the requirement of parasitic invasion of the brain itself. In the present report, we experimentally test these divergent predictions.

## Materials and Methods

### Animals

Male adult Wistar rats were used, procured from the National University of Singapore (>8 weeks at the start of the experiment). All experimental procedures were reviewed and approved by local institutional animal use and care committee. Standard laboratory animal housing conditions were employed (12:12 light-dark cycle; lights on at 07:00 h; ad libitum food and water). The number of animals was estimated based on behavioral variance observed during a previous study with similar experimental procedures. All animals survived experimental procedures until the sacrifice.

### Testosterone Supplementation Experiment

Rats were treated with prophylactic antibiotic and peripherally acting analgesia 15 min prior to surgery. Surgery was performed using aseptic techniques under isoflurane anesthesia (2.5% gaseous isoflurane with pure O_2_). Testes and vas deferens were bilaterally removed through a medial incision in the scrotum. Bilateral intra-cerebral cannulas were also implanted dorsal to posterodorsal division of the medial amygdala (AP = −3.0, L = ± 3.8, v = −7.0) (18). Osmotic pumps were placed subcutaneously and connected thorough cannulas to supply either testosterone (25mM ethanol stock solution diluted to 3% in artificial cerebrospinal fluid) or placebo (3% ethanol solution in artificial cerebrospinal fluid) to the medial amygdala (ALZET Osmotic Pumps, USA). Body weights were recorded for seven days post-operatively, with no further experimental procedures planned during this period.

Aversion to bobcat urine was measured after 12 successive days of supplementation. Animals were first habituated to the testing arena (two rectangular arms of 76 cm × 9 cm each, connected by a central junction of 9 cm × 9 cm); for two consecutive days in the absence of odor. Subsequently, rats were placed in the arena pre-seeded with bobcat urine and rabbit urine in two opposing corners of the arena (2 ml, trial duration = 20 min).

The correct placement of the cannula was retrospectively confirmed using histological examination. Animals were sacrificed through transcardial perfusion of 4% paraformaldehyde dissolved in phosphate-buffered saline. Harvested brains were equilibrated in 30% (wt/vol) sucrose. Coronal sections through the medial amygdala were obtained at 40-μm thickness at −22°C (Leica CM1950 cryostat). Slide-mounted sections were examined at 400X after Nissl staining (19). One animal exhibiting incorrect placement in both hemispheres was excluded from statistical analysis. Five animals with incorrect placement in only one of the hemispheres were not excluded. Animals with unilateral placements within the medial amygdala in were not excluded due to lack of *a priori* expectation of lateralization and anatomic separation from other known androgen-responsive neuronal populations. Cannula tracks remained invisible in two animals; these animals were included in the analysis.

### Castration Experiment

A type 2 *Toxoplasma* strain, Prugniaud, was used for the infection at a dose of 5 million lab-grown tachyzoites per animals (*i.p.*). Corresponding control animals were injected with sterile buffered saline. Routine management of animals and parasites was similar to earlier studies (7). All animals were castrated >6 weeks post-infection. Surgery was performed under deep anesthesia achieved by 2% isoflurane gas mixed in pure O_2_. A medial scrotal incision was made, testes were bilaterally removed along with vas deferens, and blood vessels supplying the testis were cauterized. The scrotal incision was closed using wound clips, which were removed after 1 week. Animals were monitored daily for 1 week, and no experimental procedures were planned during this period.

Aversion to cat odor was measured ten days post-castration. Brains were harvested by decapitation immediately after behavioral testing. Posterodorsal medial amygdala was microdissected from harvested brains and genomic DNA was isolated using DNeasy Blood and Tissue Kit (Qiagen). The methylation-sensitive restriction enzyme (HpaII, New England Biolabs, USA) digestion assay was used to quantify the level of methylation of the AVP promoter in the genomic DNA, using methods described before (7). The following primers were used to estimate DNA abundance of AVP promoter site: forward GTAGACCGCCACACCTGA and reverse CCAGACATTGGTGTGTGACC.

### Data Analysis

The normality of the data was tested using the Shapiro-Wilk test **(** 20 **)**. The assumption of normality was found to be void in case of escape latency from bobcat odor for testosterone supplementation experiments. This set of data was analyzed using unpaired Student’s t-test before and after rank transformation, with a similar outcome with respect to the probability of type 1 error. Values for non-transformed data are reported. All other endpoints were determined to normally distribute and unpaired Student’s t-test was used to calculate *p* values. The effect size was calculated using Cohen’s d.

## Results

### Exogenous Testosterone Within the Medial Amygdala Recapitulated the Behavioral Effects of the Infection

**Figure 1:**
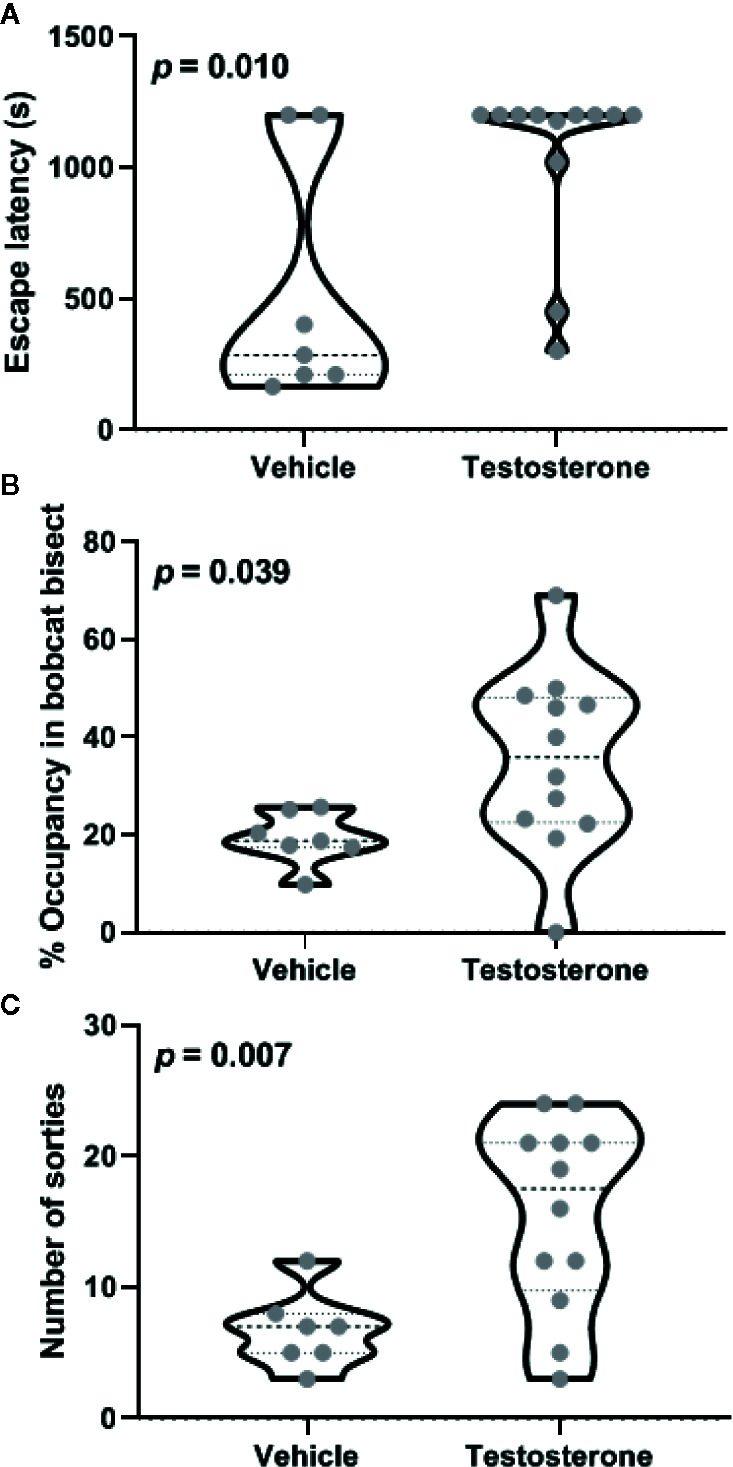
Effects of testosterone supplementation within the posterodorsal medial amygdala on defensive behavior. Latency to escape from the testing arena **(A)**, relative occupancy in bisect of the arena containing bobcat urine **(B)**, and total number of sorties made during the trial **(C)** are depicted. Violin plots in these panels depict median and inter-quartile range along with raw values for all data points (n = 7 animals for vehicle and 11 for the testosterone-treated group). The *p*-values for inter-group differences are depicted in each panel.

Castrated animals chronically supplemented with testosterone within the posterodorsal medial amygdala were compared with vehicle-treated animals for aversion to bobcat urine. Five out of seven vehicle-treated animals and four out of twelve testosterone-treated animals successfully escaped from the arena before completion of 1200-s-long trial. Inter-group comparison revealed that testosterone treatment significantly enhanced escape latency (**Figure 1A**; independent sample t-test: t_17_ = 2.90, *p* = 0.01). Analysis of the effect size demonstrated a robust difference due to testosterone treatment (mean difference: ׀∆x̅Ι = 521 ± 180 s; effect size: Cohen’s d = 1.30). Both experimental groups exhibited a significant aversion to the cat odor as demonstrated by reduced occupancy of bisect containing cat urine (**Figure 1B**; one-sample t-test against a chance expectation of 50%; t_6_ = 15.11 for placebo and t_11_ = 2.76 for testosterone, *p* < 0.02). Inter-group comparison demonstrated significant reduction in aversion to cat odor due to testosterone treatment (t_17_ = 2.24, *p* = 0.039; ׀∆x̅Ι = 16.1 ± 7.2%; Cohen’s d = 1.19). Robustness of testosterone treatment on all three endpoints measured here is borne out by the high magnitude of effect size (d > 1). Moreover, the 25^th^ percentile of the testosterone-treated group was placed well above the median of placebo-treated animals in terms of both the number of sorties made and time spent near cat urine. Testosterone treated animals also conducted more sorties to vicinity of the cat odor (**Figure 1C**; t_17_ = 3.06, *p* = 0.007; ׀∆x̅Ι = 8.9 ± 2.9; Cohen’s d = 1.61).

**Figure 2:**
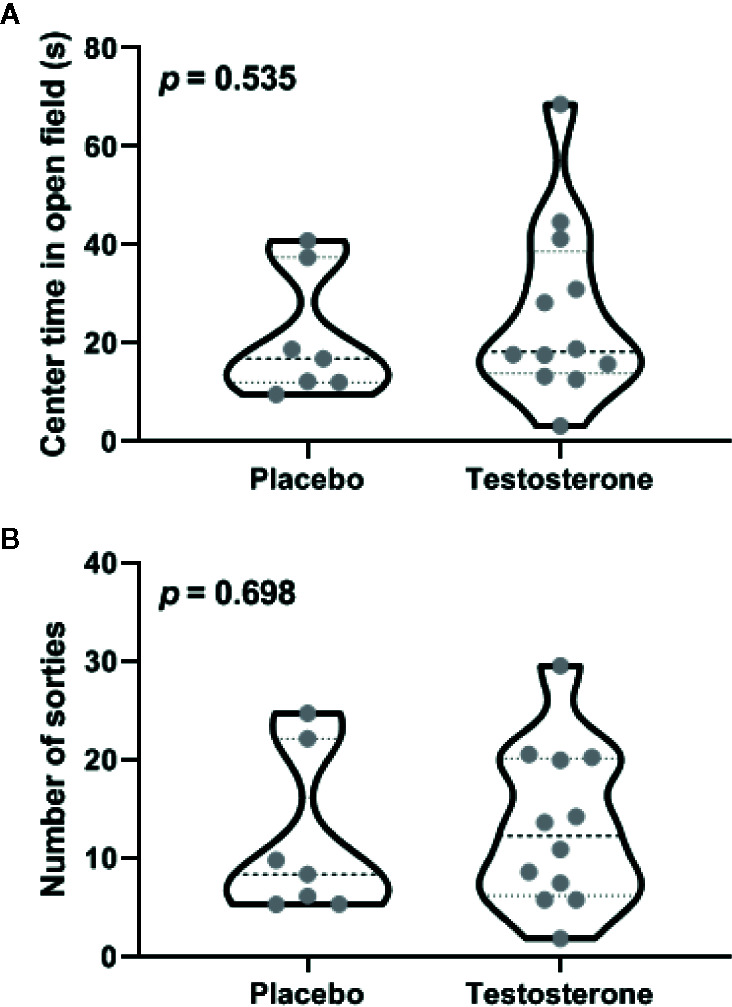
Effects of testosterone supplementation within the posterodorsal medial amygdala on anxiety measured in an open-field arena. The percentage of time spent in the center of the arena **(A)** and total number of sorties made during the trial **(B)** are depicted. Violin plots in these panels depict median and inter-quartile range along with raw values for all data points (n = 7 animals for vehicle and 11 for the testosterone-treated group). The *p*-values for inter-group differences are depicted in each panel.

When tested for anxiety in an open field, both groups of animals exhibited comparable avoidance of anxiogenic center (**Figure 2A**; t_17_ = 0.634, *p* = 0.535; ׀∆x̅Ι = 4.9 ± 7.8 s; Cohen’s d = 0.32) and also in number of sorties made during the trial (**Figure 2B**; t_17_ = 0.394, *p* = 0.698; ׀∆x̅Ι = 1.5 ± 3.9; Cohen’s d = 0.12). Thus, effects of testosterone supplementation on innate aversion within medial amygdala did not generalize to anxiety-like behaviors tested in the open field.

### Behavioral and Molecular Effects of the Infection Were Obliterated by Castration

**Figure 3:**
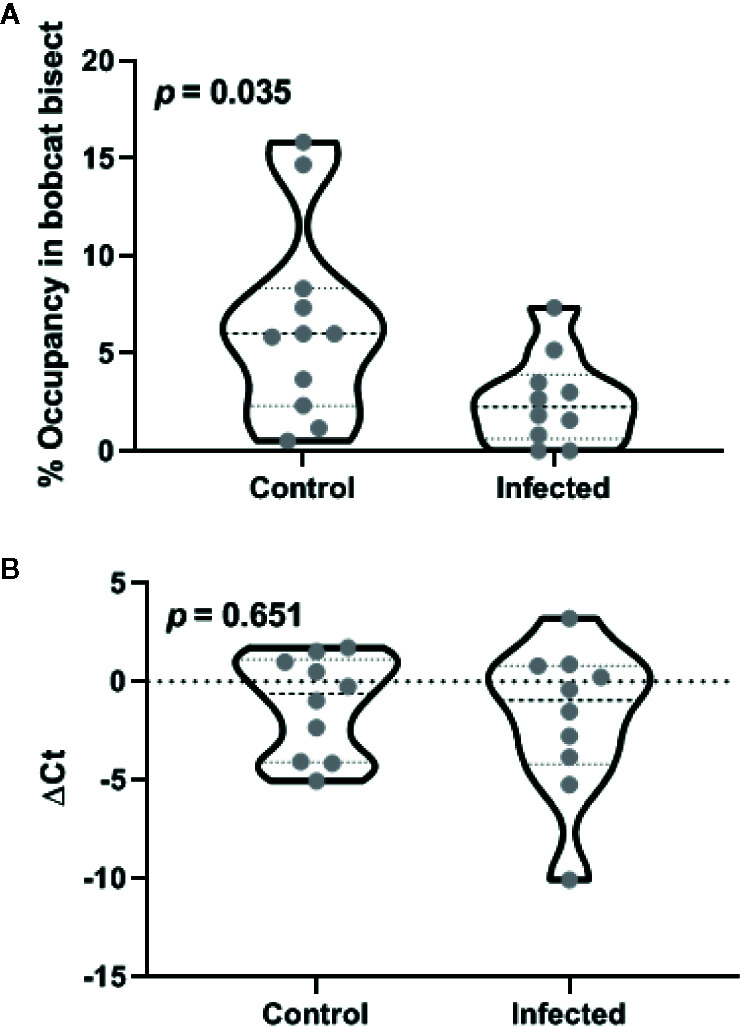
Effects of castration after the chronic phase of *Toxoplasma gondii* infection on defensive behavior **(A)** and medial amygdala arginine vasopressin promoter methylation **(B)**. Violin plots in these panels depict median and inter-quartile range along with raw values for all data points (n = 11 animals for control and 12 for the infected group). The *p*-values for inter-group differences are depicted in each panel.

Both control and infected animals exhibited a significant aversion to the cat odor as demonstrated by reduced occupancy of bisect containing cat urine (**Figure 3A**, one-sample t-test against the chance expectation of 50%; t_10_ = 28.92 for control and t_9_ = 64.76 for testosterone, *p* < 0.0001). In contrast to reduced aversion in gonad-intact animals, *Toxoplasma gondii* infection significantly increased aversion to cat urine in the castrates (t_19_ = 2.27, *p* = 0.035; ׀∆x̅Ι = 3.9 ± 1.7%; Cohen’s d = 1.00). Seventy-fifth percentile of the infected group was observed to be placed well below the median of the control group, suggesting a robust decrease in occupancy near cat odor due to *Toxoplasma gondii*.

DNA methylation within AVP promoter was quantified using a methylation specific endonuclease treatment and subsequent quantitative PCR. Endonuclease treatment did not produce statistically significant departure from theoretical expectation based on complete methylation (**Figure 3B**; one-sample t-test against zero ∆Ct; t_9_ = 1.51 for control and t_9_ = 1.56 for testosterone, *p* > 0.15). *Toxoplasma gondii* infection did not result in statistically significant change in the methylation status (t_18_ = 0.46, *p* = 0.651; ׀∆x̅Ι = 0.67 ± 1.4 cycles; Cohen’s d = 0.10). Lack of infection effect *vis-à-vis* controls was also demonstrated by low probability of type 2 error (observed power = 0.93 at α = 0.05).

## Discussion

*Toxoplasma gondii* has been demonstrated to invade both the brain and testes of the infected male rats (7). Data presented in this report suggest the invasion of testes and the resultant increase in testosterone is critical for the purported behavioral manipulation. *Toxoplasma gondii* invades host testes and increases testosterone production (6). Greater testosterone (or its estrogen metabolites) then causes epigenetic modification in the promoter for AVP within the medial amygdala (7). This results in greater recruitment of the medial amygdala AVP system. These neurons are typically recruited during the sexual approach, e.g., during the processing of female derived pheromones by male rats (7). Thus, the atypical increase in medial amygdala AVP leads to a “misassignment” of semiochemical valence during cat odor exposure (12).

This model reconciles earlier observation that attenuated strains of *Toxoplasma* do not cause sustained encystment in the brain but do cause loss of predator aversion (21). Current observations build evidence for the possibility that effects of *Toxoplasma* on the neural processing result from endocrine effects of gonads rather than paracrine effects arising from the physical presence of the parasite within brain tissue. There are two important caveats that are worth considering. Firstly, several papers have reported localized effects of *Toxoplasma* cysts on neurotransmitter processing (22, 23). The relatively long-distance signaling proposed here will need to be reconciled with these observations. Secondly, female rodents exhibit analogous behavioral changes without high levels of endogenous testosterone (24). Further experiments are needed to examine if dimorphic neuroendocrine mediators underlie uni-morphic effects of Toxoplasma. Our data also do not measure the relative contribution of testosterone binding to androgen receptors or its aromatized metabolites binding to estrogen receptors.

We have earlier reported that *Toxoplasma gondii* infection in castrated rats does not lead to loss of host aversion to cat odors (6). Yet those prior observations left the causal links ambivalent. Testosterone is an immune-suppressive steroid. It is thus plausible that castration before the infection altered the potency of the immune system and interfered with the establishment of the chronic infection (25). In contrast, the present study employed animals that were gonad-intact during the acute phase of the infection. This treatment did not interfere with acute inflammation and the establishment of chronic infection *per se*. We further demonstrate recapitulation of the behavior in castrated rats using selective re-supplementation of testosterone within the medial amygdala of the brain.

Testosterone is generally thought to be anxiolytic (26). But, its role in reducing innate fear remains under-explored, with the exception of a single paper reporting that castration enhances freezing in response to a synthetic analog of fox odor (27). Current observations provide evidence that testosterone acting within the medial amygdala is sufficient to reduce innate fear. Many neurons in rat medial amygdala express AVP in a testosterone dependent manner (28). We have earlier shown that these neurons are selectively recruited during the processing of sexual pheromones in rats (7). Others have shown that lesions of medial amygdala reduce aversion to cat odors (29). Thus, the medial amygdala is essential for processing semiochemical information pertaining to both defensive and reproductive behaviors. Androgenic milieu within the medial amygdala can change the balance between opposing valence of reproductive and defensive semiochemicals.

*Toxoplasma gondii* infection in immune-competent humans is associated with sequelae of personality differences that show sexual dimorphism (30). Furthermore, case-control studies report that the infection increases circulating testosterone levels in male but not in female human volunteers (31). Observations in the present report suggest that testosterone might mediate some of the observed sex-dependent personality differences. While *Toxoplasma gondii* infection has been widely studied in the context of host-parasite relations, this model system also affords a perturbation system to study the neurobiology of fear. Innate aversion to predator odors is often used to model fear-related psychiatric conditions like post-traumatic stress disorders or generalized anxiety (32, 33). The present results suggest that interaction between androgens and the brain can provide a moderating influence on fear-related behaviors.

In conclusion, current observations suggest that downstream neural effects of gonadal steroids within the medial amygdala in male rodents are critical for host behavioral change after *Toxoplasma gondii* infection. Furthermore, these observations demonstrate that testosterone, or its metabolites, reduce fear by recruiting medial amygdala.

## Data Availability Statement

The datasets generated for this study are available on request to the corresponding author.

## Ethics Statement

The animal study was reviewed and approved by Nanyang Technological University IACUC.

## Author Contributions

AV and DS designed the experiments. SH, SA-S, and DS conducted the experiments. All authors contributed to the article and approved the submitted version.

## Conflict of Interest

The authors declare that the research was conducted in the absence of any commercial or financial relationships that could be construed as a potential conflict of interest.
