# Corrigendum: Testosterone Acts Within the Medial Amygdala of Rats to Reduce Innate Fear to Predator Odor Akin to the Effects of *Toxoplasma gondii* Infection

**DOI:** 10.3389/fpsyt.2021.735656

**Published:** 2021-09-09

**Authors:** Dhiraj Kumar Singh, Shantala Arundathi Hari Dass, Samira Abdulai-Saiku, Ajai Vyas

**Affiliations:** School of Biological Sciences, Nanyang Technological University, Singapore, Singapore

**Keywords:** androgen, behavioral manipulation, defensive behaviors, parasites, pheromones, semiochemicals

In the original article, there was an error. The testosterone concentration in section **Materials and Methods** was stated to be 25 mM. This testosterone concentration related to the stock solution instead, which was further diluted 33 times.

A correction has been made within a single line in the section **Materials and Methods**, subsection **Testosterone Supplementation Experiment**, **first paragraph**:

Osmotic pumps were placed subcutaneously and connected thorough cannulas to supply either testosterone (25 mM ethanol stock solution diluted to 3% in artificial cerebrospinal fluid) or placebo (3% ethanol solution in artificial cerebrospinal fluid) to the medial amygdala (ALZET Osmotic Pumps, USA).

The authors apologize for this error and state that this does not change the scientific conclusions of the article in any way. The original article has been updated.

## Publisher's Note

All claims expressed in this article are solely those of the authors and do not necessarily represent those of their affiliated organizations, or those of the publisher, the editors and the reviewers. Any product that may be evaluated in this article, or claim that may be made by its manufacturer, is not guaranteed or endorsed by the publisher.

